# Codon Usage Bias and Cluster Analysis of the *MMP-2* and *MMP-9* Genes in Seven Mammals

**DOI:** 10.1155/2022/2823356

**Published:** 2022-09-05

**Authors:** Tanliang Ouyang, Jincheng Zhong, Zhixin Chai, Jiabo Wang, Ming Zhang, Zhijuan Wu, Jinwei Xin

**Affiliations:** ^1^Key Laboratory of Qinghai-Tibetan Plateau Animal Genetic Resource Reservation and Utilization, Southwest Minzu University, Chengdu 610225, China; ^2^State Key Laboratory of Hulless Barley and Yak Germplasm Resources and Genetic Improvement, Lhasa 850000, China

## Abstract

Matrix metalloproteinase (MMP)-2 and MMP-9 are a family of Zn^2+^ and Ca^2+^-dependent gelatinase MMPs that regulate muscle development and disease treatment, and they are highly conservative during biological evolution. Despite increasing knowledge of *MMP* genes, their evolutionary mechanism for functional adaption remains unclear. Moreover, analysis of codon usage bias (CUB) is reliable to understand evolutionary associations. However, the distribution of CUB of *MMP-2* and *MMP-9* genes in mammals has not been revealed clearly. Multiple analytical software was used to study the genetic evolution, phylogeny, and codon usage pattern of these two genes in seven species of mammals. Results showed that the *MMP-2* and *MMP-9* genes have CUB. By comparing the content of synonymous codon bases amongst seven mammals, we found that *MMP-2* and *MMP-9* were low-expression genes in mammals with high codon conservation, and their third codon preferred the G/C base. RSCU analysis revealed that these two genes preferred codons encoding delicious amino acids. Analysing what factors influence CUB showed that the third base distributors of these two genes were C/A and C/T, and GC_3S_ had a wide distribution range on the ENC plot reference curve under no selection or mutational pressure. Thus, mutational pressure is an important factor in CUB. This study revealed the usage characteristics of the *MMP-2* and *MMP-9* gene codons in different mammals and provided basic data for further study towards enhancing meat flavour, treating muscle disease, and optimizing codons.

## 1. Introduction

Codon usage bias (CUB) is defined as unequal utilisation in the frequency of synonymous codons in coding amino acids (AAs), and it has been used extensively for investigating gene phylogeny [1]. The synonymous codon characteristics include universality, degeneracy, and wobble, and they should be used randomly to encode corresponding AAs with no pressure of interference pressure. However, CUB can be affected by nucleotides composition, translation, hydrophobicity, tRNA abundance, and protein structure [2–6]. Notably, natural selection and mutational pressure, which drive the correct translation process, are the major factors associated with CUB [7, 8]. Natural selection affects the pattern of codon usage in organisms, and mutational pressure may arise whilst the proportion of codon bases changes. CUB greatly increases the variability of genetic information and reflects the genetic drift of codons to a certain extent [9]. Therefore, CUB can reveal the evolution of genes or organisms and environmental adaptation [10].

CUB is assessed by using the effective number of codons (ENC), codon adaptation index (CAI), frequency of optimal codons (FOP), codon bias index (CBI), and relative usage of synonymous codons (RSCU). ENC is calculated by comparing the GC content of synonymous codon positions [11]. CAI is 0-1; the closer the value is to 1, the stronger the nucleotide bases prefer synonymous codons [12]. FOP and CBI are both 0-1. These two indicators are close to 1, and the optimal codon for encoding amino acids is preferred. However, if CBI is negative, the optimal codon usage is less than the average number of codons used [3, 13]. RSCU is the specific value between the actual observation and theoretical observation, amongst which the theoretical observation value is the observation value when the synonymous codon usage frequency is the same, namely, there is no codon bias. If RSCU = 1, there is no CUB. If RSCU > 1, the appearance frequency of the codon is higher than the other synonymous codon. By contrast, it indicates lower genes. If RSCU > 2, then the frequency of CUB is extremely high [14].

Matrix metalloproteinases (MMPs) are a family of Zn^2+^ and Ca^2+^-dependent proteolytic enzymes that are widely expressed in animal tissues and highly conservative during biological evolution [15]. *MMP-2* and *MMP-9* can regulate muscle growth, repair, and some relative processes that affect biochemical reactions for muscle regulation [16]. Although recent research mainly focused on exploring *MMP-2* and *MMP-9* function for animal skeletal muscle development, healing diseased muscle and even meat [17–23], studies on MMP codons is rare. Therefore, there is an urgent need for exploring mammals' *MMP-2* and *MMP-9* genetic evolution and codon usage pattern regulating muscle growth.

In this study, seven mammals (*Bos grunniens*, *Bos taurus*, and *Sus scrofa* among Artiodactyla; *Macaca mulatta* in Primates; *Canis lupus familiaris* in Carnivora; *Oryctolagus cuniculus* in Lagomorpha; and *Mus musculus* in Rodentia) were chosen to analyse CUB and base pair composition dynamics. This study would give insight into the factors affecting CUB for *MMP-2* and *MMP-9* genes and provide basic data for enhancing the meat flavour and finding a promising gene treatment for muscle disease.

## 2. Materials and Methods

### 2.1. Software

MEGA 7.0, CodonW 1.4.2, pheatmap, and ggplot packages based on R 4.4.3 software were used to complete the relevant analysis.

### 2.2. Base Composition of MMP Genes' CDS in Different Mammals

**Figure 1:**
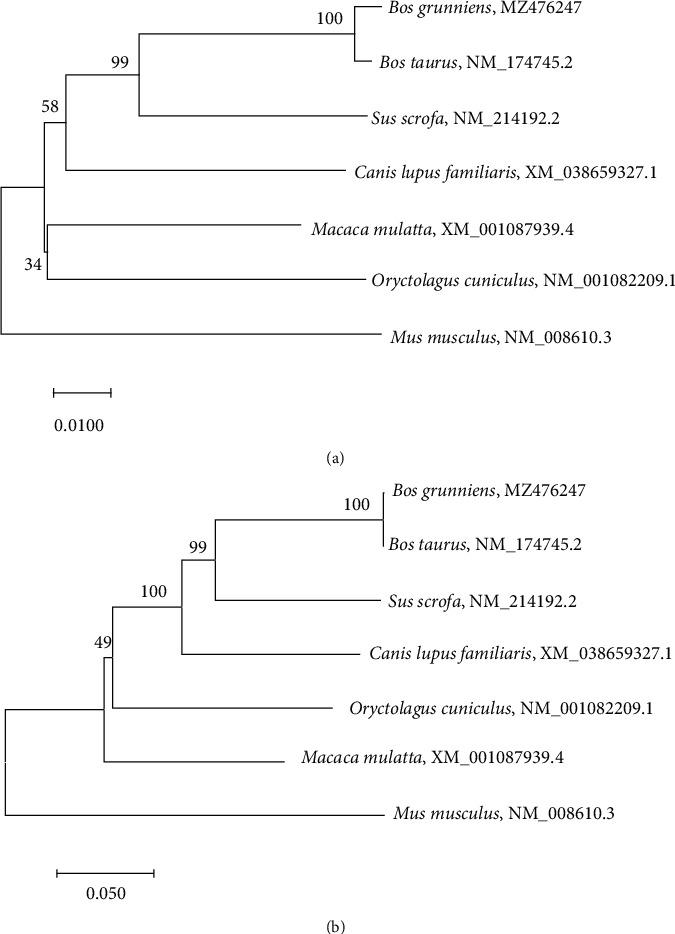
NJ trees of *MMP-2* (a) and *MMP-9* (b) genes of seven mammal species.

The coding sequence (CDS) of yak *MMP-2* and *MMP-9* genes were obtained in our laboratory, and the NCBI accession numbers were MZ476247 and MZ476248, respectively. The CDS of other animals' genes were from NCBI GenBank, and their accession numbers are shown in Figure 1.

CodonW 1.4.2 software developed by J. Peden was used to analyse the *MMP-2* and *MMP-9* CDS in seven mammals for calculating *A*/*T* (*A*/*T* base content, the same below), *G*/*C*, T_3S_ (third base of the codon is *T* content, the same below), C_3S_, A_3S_, G_3S_, GC_3S_, AT_3S_, ENC, CAI, CBI, FOP, and RSCU [24]. R packages pheatmap and ggplot2 were used to analyse the data.

### 2.3. PR2 Plot

PR2 plot could analyse the bias amongst ATCG under gene mutation [25]. If the frequency of the third base is *A* > *T*, then dots are scattered on the top of the PR2 plot. If the frequency is *C* > *G*, then dots are on the left. When the codon does not show usage bias, the dots are in the centre of the graph [26].

### 2.4. Codon Neutral Analysis

Codon neutral analysis was carried out by the correlation analysis of GC_12_ (the average of the GC content of the first and second bases) and GC_3S_ to compare the influence of natural selection pressure and mutational pressure on CUB [27]. A significant correlation between GC_12_ and GC_3S_ indicated that mutational pressure had a strong influence on codon preference; otherwise, natural selection influenced CUB [28].

### 2.5. ENC Plot

The relationship between ENC and GC_3S_ without environmental selection pressure could be simulated by the following formula (1). The ENC/GC_3S_ reference curve shows the main characteristics of codon usage patterns [24]. If CUB is more affected by natural selection, it should be below the standard curve. By contrast, it should be above the standard curve if it is more affected by other factors such as gene mutation. In general, the ENC is from 35 to 61. If ENC > 35, CUB is weak [11].

(1) $$\begin{array}{c} \text{ENC}=2+{\text{GC}}_{3S}+\frac{29}{{\left({\text{GC}}_{3S}\right)}^{2}+{\left(1-{\text{GC}}_{3S}\right)}^{2}}. \end{array}$$

## 3. Results

### 3.1. Phylogenetic Analysis

Neighbour joining (NJ) trees were established based on the *MMP-2* and *MMP-9* CDS in seven mammals. The results (Figure 1) showed that the *MMP-2* and *MMP-9* genes of *Bos grunniens* were similar to those of *B. taurus*. These two genes of *S. scrofa* were similar to those of *B. grunniens* and *B. taurus*. Interestingly, the *MMP-9* genes of *C. lupus familiaris* showed closer proximity to those of *S. scrofa* but those of the *MMP-2* gene was farther.

### 3.2. Nucleotide Composition of MMP-2 and MMP-9 Genes

Compared with the content of codon bases of the *MMP-2* and *MMP-9* genes in seven mammals, the results showed (Tables 1 and 2) that the *G*/*C* content was higher than the *A*/*T* content. Most mammals' *MMP-2* and *MMP-9* GC_3S_ were larger than AT_3S_, except for the *MMP-2* gene of *B. taurus* and *M. musculus*. The above findings indicated that the *MMP-2* and *MMP-9* gene codons preferred GC_3S_.

The codon usage results (Tables 3 and 4) showed that ENCs of the *MMP-2* and *MMP-9* genes in seven mammals were 40–56, indicating that these two genes had low expression and their codon conservation was high.

CAI showed that the preference for synonymous codons of the *MMP-9* genes in seven mammals was significantly better than that of *MMP-2*, but both were lower than 0.3, indicating that it failed to reflect the preference of synonymous codons.

FOP and CBI results of the *MMP-2* and *MMP-9* genes showed that the optimal codon usage of *MMP-2* in *B. grunniens* and *B. taurus* was inferior to the five other animals, whilst the optimal codon usage of *MMP-9* was better than that of *MMP-2*.

### 3.3. RSCU Analysis

The RSCU results of the *MMP-2* and *MMP-9* genes showed that these two genes had a preference for 27 and 20 codons, respectively (Tables 5 and 6). Amongst them, CUG (encoding leucine, Leu) of *MMP-2*, CUG (encoding leucine, Leu), ACC (encoding threonine, Thr), and CGC (encoding arginine, Arg) of *MMP-9* had strong high CUB (RSCU > 2).

**Figure 2:**
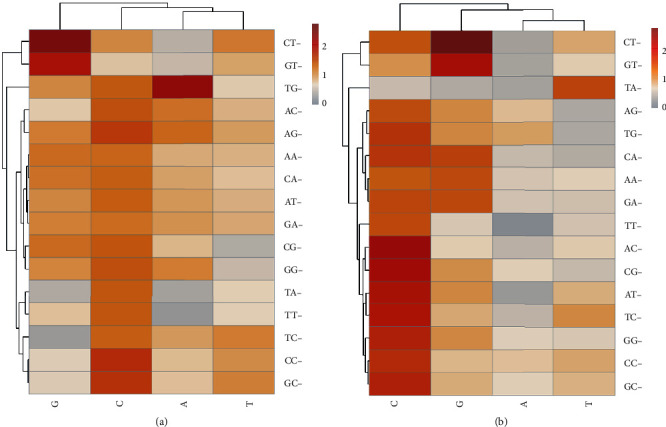
Heat maps of *MMP-2* (a) and *MMP-9*. (b) Correlation coefficient of codons with GC_3S_.

Heat map analysis of the correlation between codon base composition and GC_3S_ (Figure 2) showed that most of the codons of the *MMP-2* and *MMP-9* genes in different mammals were positively correlated with GC_3S_ and in line with AC-, CG-, AT-, TC-, GG-, CC-, GC-, and other codons whose third base was C.

**Figure 3:**
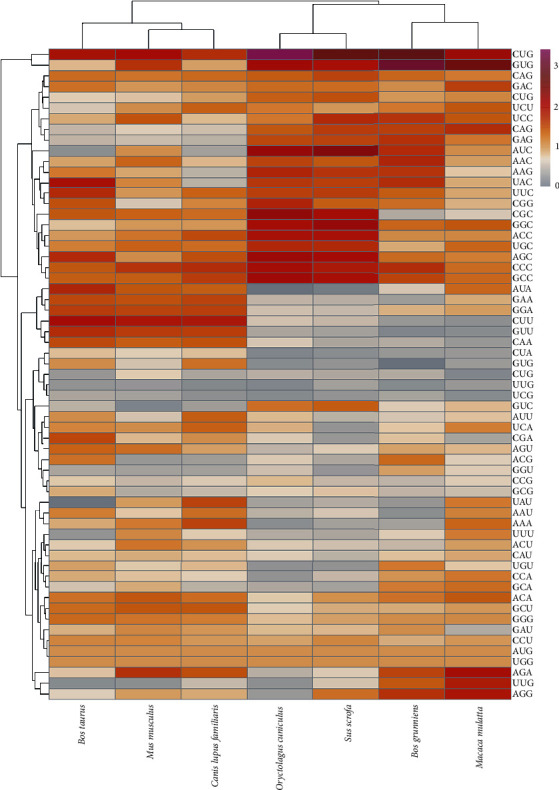
Clustering of RSCU values of each codon in *MMP-2* gene.

**Figure 4:**
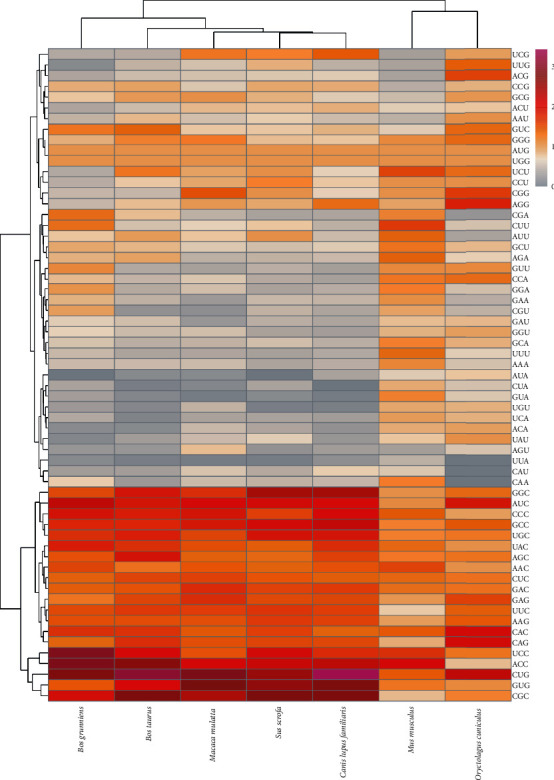
Clustering of RSCU values of each codon in *MMP-9* gene.

Cluster analysis of the RSCU of the *MMP-2* and *MMP-9* genes showed that the *MMP-2* gene preferred CUG, GUG, UCC, GAG, AUC, AAC, UAC, GCC, AGA, UUG, and AGG codons, which were mainly involved in encoding Leu (leucine), Val (valine), Ser (serine), Glu (glutamic acid), Iso (isoleucine) Asn (asparagine), Tyr (tyrosine), Gly (glycine), and Arg (arginine), respectively (Figures 3 and 4). In addition to *B. taurus* and *C. lupus familiaris*, the five other species had a strong preference for CUG and GUG (RSCU > 2), amongst which the RSCU of *O. cuniculus* and *B. grunniens* > 3. The *MMP-9* gene preferred UCC, ACC, CGC, CUG, and AUC codons, which are mainly involved in Ser, Thr, Arg, Leu, and Iso, respectively. Except for *M. musculus* and *O. cuniculus*, the last five species had strong preferences similar to one another, indicating that the *MMP-9* gene was more conservative than *MMP-2*.

### 3.4. Factors Influenced CUB

**Figure 5:**
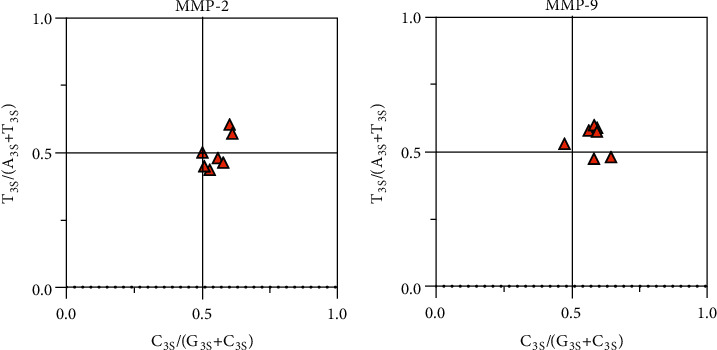
PR2 plot among seven species.

The PR2 plot result (Figure 5) showed that the ATCG base distribution of the *MMP-2* and *MMP-9* genes amongst seven mammals was above 0.5 on the *x*-axis. The bases distribution of the *MMP-2* genes was mainly on the *x*-axis and the upper right of the *y*-axis and that of the *MMP-9* genes was to the *x*-axis and the upper right of the *y*-axis. The above results indicated that the contents of A_3S_ and C_3S_ for the *MMP-2* gene and the content of T_3S_ and C_3S_ for the *MMP-9* gene were high, respectively.

**Figure 6:**
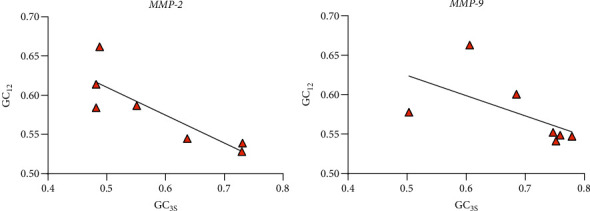
Codon neutral analysis.

Neutral analysis (Figure 6 and Table 7) showed that GC_3S_ of these two genes was in the range of 0.44–0.78, whereas GC_12_ was from 0.52 to 0.67. The difference was that GC_12_ and GC_3S_ of the *MMP-2* gene were strongly negatively correlated (Pearson *r* = −0.851, *p* value < 0.05), whilst GC_12_ and GC_3S_ of the *MMP-9* gene were not significantly correlated, indicating that the base composition of the *MMP-2* gene codons was susceptible to mutational pressure, but the factor influencing the *MMP-9* gene was natural selection.

**Figure 7:**
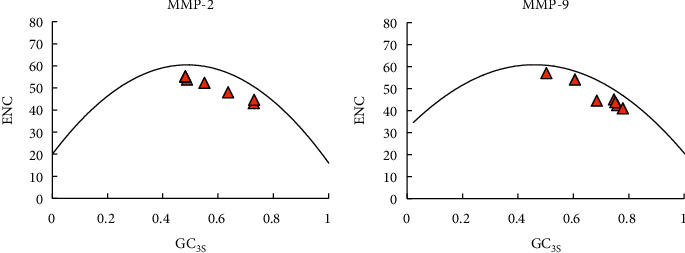
ENC plot among seven species.

The ENC plot showed (Figure 7 and Table 7) that all ENC/GC_3S_ dots of the *MMP-2* and *MMP-9* genes were distributed below the reference line. ENC and GC_3S_ had a strongly negative correlation (*MMP-2*: Pearson *r* = −0.993, *p* value < 0.01; *MMP-9*: Pearson *r* = −0.963, *p* value < 0.01), and the distribution range of GC_3S_ was large, indicating that the CUB of these two genes was affected by mutational pressure.

## 4. Discussion

This study found that gelatinase *MMP* genes had CUB for encoding amino acids such as Ile, Arg, Glu, and Ser related to muscle development and meat quality. Gly, Arg, and Leu can promote collagen synthesis, and animal muscle is the main way to obtain natural collagen for humans [29, 30]. Delicious amino acids (DAAs), including Glu, Gly, Ser, Asp, Arg, and Ile, are known as precursor substances that determine the flavour of meat and can improve the taste of chicken and keep the meat soft [31]. Recent research found that the quality of chicken improves and the content of DAAs increases [32]. Otherwise, Strecker amino acids (SAAs), including Phe (phenylalanine), Cys (cysteine), Ile (isoleucine), and Leu (leucine), are highly related to the production of flavour. The higher their content, the stronger the fragrance [33]. For the *MMP-2* and *MMP-9* genes, the RSCUs of AUC encoding Ile; UCC and AGC encoding Ser; CGC encoding Arg; GAC encoding Asp; GAG encoding Glu; UUC encoding Phe; and GGA, GGC, and GGG encoding Gly were > 1. In particular, the RSCUs of CUG encoding Leu and CGC encoding Arg > 2; this value indicated that *MMP-2* and *MMP-9* demonstrated CUB for DAAs and SAAs. Besides, Leu, Ile, and Val belong to branched-chain amino acids (BCAAs), and they are essential AAs in humans and animals, accounting for about 35% of muscle protein. Previous studies have found that skeletal muscle, as the initial site of BCAAs catabolism, can be activated by branched-chain keto acids (BCKAs) to increase BCAAs synthesis to relieve muscle wasting disorders [34]. Also, Leu supplementation could be the prevention and treatment of sarcopenia with aging [35]. Thus, BCAAs are important regulators of metabolism and metabolic health in *in vivo* [36]. The gelatinase MMP CUB associated with corresponding AAs can provide basic data for the improvement of meat quality and muscle disease of MMP molecular modification.

Mutational pressure may be the main factor influencing the CUB of MMPs. This study found that the clustering results of the RSCU were different from the NJ trees of the genes, indicating that the MMP genes were highly conserved but maybe subjected to mutations during the evolution of different species. This influence caused a decline in the accuracy of single-gene species classification. Nucleotide AT (U) CG base composition is an important feature of genes, and the GC content can reflect the overall trend of gene mutation which is a decisive factor affecting the frequency of nucleotide use. Changes in the third base of the codon did not affect the encoded AAs, so GC_3S_ could be an important reference for analysing the codon usage pattern. The gene mutation will affect the composition of the synonymous codon third bases with no natural selection, and the stronger the CUB, the more the codon is inclined to GC_3S_ [37, 38]. Novembre et al. also found that the third base distribution of the *MMP-2* and *MMP-9* genes is mainly AC_3S_ and CT_3S_, respectively, and the ENC/GC_3S_ dot distribution can reach a wide range compared with the reference curve with gene mutation pressure. Thus, mutational pressure may play an important role in affecting the CUB for *MMP-2* and *MMP-9* genes, which also explains the difference in RSCU clustering in the seven mammals.

Interestingly, we also found that the clustering results based on the RSCU of the *MMP-2* gene were not completely consistent with the phylogenetic results based on the *MMP-2* gene's CDS. Given that wild yak and Tibetan antelope grow in harsh environments with low altitudes and oxygen consumption, their *EGLN1* gene has mutated changing nucleotide bases and leading to CUB changes [39, 40]. Therefore, we believe that the phylogenetic evolution of *MMP-2* genes should not only refer to gene sequence but also CUB, which could be a supplement to species classification.

## 5. Conclusion

*MMP-2* and *MMP-9* are low-expression genes in mammals, and their codons are highly conservative. Both have a CUB at GC_3S_ and prefer codons encoding DAAs and SAAs for improving soft meat and muscle disease treatment.

## Figures and Tables

**Table 1:** Nucleotide composition in the sequence of *MMP-2* gene.

Species	*A*/*T* (%)	*G*/*C* (%)	*T *_3S_ (%)	*C *_3S_ (%)	*A *_3S_ (%)	*G *_3S_ (%)	GC_3S_ (%)	AT_3S_ (%)
*Bos grunniens*	0.415	0.585	0.2093	0.372	0.2081	0.3703	0.637	0.363
*Bos taurus*	0.384	0.616	0.2638	0.3049	0.34	0.2724	0.488	0.512
*Macaca mulatta*	0.409	0.591	0.2247	0.3128	0.2741	0.3031	0.551	0.449
*Mus musculus*	0.433	0.567	0.2974	0.3234	0.3212	0.2566	0.482	0.518
*Oryctolagus cuniculus*	0.4	0.6	0.1997	0.5452	0.1304	0.3621	0.73	0.27
*Sus scrofa*	0.393	0.607	0.1871	0.5485	0.1404	0.3488	0.731	0.269
*Canis lupus familiaris*	0.417	0.583	0.284	0.3302	0.3285	0.2415	0.482	0.518

**Table 2:** Nucleotide composition in the sequence of *MMP-9* gene.

Species	*A*/*T* (%)	*G*/*C* (%)	*T *_3S_ (%)	*C *_3S_ (%)	*A *_3S_ (%)	*G *_3S_ (%)	GC_3S_ (%)	AT_3S_ (%)
*Bos grunniens*	0.366	0.634	0.1842	0.5263	0.1981	0.2891	0.685	0.315
*Bos taurus*	0.378	0.622	0.173	0.5554	0.1202	0.3781	0.759	0.241
*Macaca mulatta*	0.379	0.621	0.1766	0.5135	0.1277	0.3978	0.747	0.253
*Mus musculus*	0.433	0.567	0.2776	0.3436	0.3056	0.2458	0.503	0.497
*Oryctolagus cuniculus*	0.345	0.655	0.2237	0.3099	0.1976	0.3431	0.606	0.394
*Sus scrofa*	0.385	0.615	0.1814	0.5411	0.1207	0.3852	0.752	0.248
*Canis lupus familiaris*	0.373	0.627	0.1554	0.5702	0.1143	0.39	0.779	0.221

**Table 3:** Codon analysis of *MMP-2* gene.

Species	ENC	CAI	CBI	Fop	GC_1S_ (%)	GC_2S_ (%)	GC_12_ (%)
*Bos grunniens*	48.04	0.193	−0.004	0.416	0.4932	0.5961	0.54465
*Bos taurus*	53.82	0.129	−0.006	0.386	0.7471	0.576	0.66155
*Macaca mulatta*	52.31	0.157	0.006	0.413	0.4982	0.6751	0.58665
*Mus musculus*	55.09	0.163	0.034	0.416	0.6238	0.5439	0.58385
*Oryctolagus cuniculus*	43.1	0.29	0.193	0.539	0.565	0.491	0.528
*Sus scrofa*	44.62	0.261	0.17	0.524	0.5579	0.52	0.53895
*Canis lupus familiaris*	55.29	0.146	0.014	0.4	0.6706	0.5569	0.61375

**Table 4:** Codon analysis of *MMP-9* gene.

Species	ENC	CAI	CBI	Fop	GC_1S_ (%)	GC_2S_ (%)	GC_12_ (%)
*Bos grunniens*	44.44	0.256	0.24	0.558	0.6713	0.5295	0.6004
*Bos taurus*	42.48	0.291	0.249	0.566	0.6015	0.4955	0.5485
*Macaca mulatta*	44.99	0.252	0.191	0.53	0.5997	0.5045	0.5521
*Mus musculus*	56.96	0.16	0.042	0.419	0.6105	0.5448	0.57765
*Oryctolagus cuniculus*	54.05	0.154	0.011	0.419	0.5065	0.8191	0.6628
*Sus scrofa*	43.79	0.282	0.222	0.549	0.5958	0.4863	0.54105
*Canis lupus familiaris*	40.95	0.287	0.243	0.563	0.6025	0.4916	0.54705

**Table 5:** RSCU for *MMP-2* gene among seven species.

AA	Codon	Frequency	RSCU
Phe	UUU	66	0.675714
	UUC^*∗*^	142	1.324286

Leu	UUA	28	0.268571
	UUG	71	0.732857
	CUU^*∗*^	127	1.09
	CUC	101	1.011429
	CUA	53	0.468571
	CUG^*∗∗*^	239	2.428571

Ile	AUU	34	0.801429
	AUC^*∗*^	67	1.28
	AUA	34	0.918571
Met	AUG	110	1

Val	GUU	65	0.861429
	GUC	47	0.715714
	GUA	39	0.522857
	GUG^*∗*^	122	1.902857

Ser	UCU^*∗*^	89	1.078571
	UCC^*∗*^	104	1.27
	UCA	70	0.904286
	UCG	23	0.317143

Pro	CCU	157	0.974286
	CCC^*∗*^	256	1.641429
	CCA	124	0.75
	CCG	102	0.635714

Thr	ACU	74	0.795714
	ACC^*∗*^	134	1.365714
	ACA^*∗*^	108	1.142857
	ACG	64	0.697143

Ala	GCU^*∗*^	128	1.047143
	GCC^*∗*^	199	1.585714
	GCA	99	0.735714
	GCG	79	0.631429

Tyr	UAU	30	0.678571
	UAC^*∗*^	75	1.321429

His	CAU	92	0.74
	CAC^*∗*^	148	1.26

Gln	CAA	144	0.864286
	CAG^*∗*^	132	1.135714

Asn	AAU	40	0.787143
	AAC^*∗*^	75	1.212857

Lys	AAA	58	0.822857
	AAG^*∗*^	138	1.178571

Asp	GAU	94	0.835714
	GAC^*∗*^	131	1.164286

Glu	GAA	116	0.944286
	GAG^*∗*^	121	1.055714

Cys	UGU	73	0.69
	UGC^*∗*^	151	1.31
Trp	UGG	206	1

Arg	CGU	34	0.428571
	CGC^*∗*^	104	1.327143
	CGA	68	0.772857
	CGG^*∗*^	92	1.177143

Ser	AGU	66	0.897143
	AGC^*∗*^	112	1.534286

Arg	AGA^*∗*^	109	1.212857
	AGG^*∗*^	93	1.08

Gly	GGU	71	0.527143
	GGC^*∗*^	207	1.371429
	GGA^*∗*^	156	1.074286
	GGG^*∗*^	149	1.03

TER	UAA	21	0.374286
	UAG	21	0.425714
	UGA^*∗∗*^	132	2.201429

*Note.* ^*∗*^RSCU > 1; ^*∗∗*^RSCU > 2; AA. amino acid; TER. termination codon; the same below.

**Table 6:** RSCU for *MMP-9* gene among seven species.

AA	Codon	Frequency	RSCU
Phe	UUU	72	0.584285714
	UUC^*∗*^	197	1.415714286

Leu	UUA	17	0.17
	UUG	44	0.621428571
	CUU	76	0.848571429
	CUC^*∗*^	107	1.362857143
	CUA	33	0.357142857
	CUG^*∗∗*^	195	2.641428571

Ile	AUU	30	0.811428571
	AUC^*∗*^	61	1.881428571
	AUA	11	0.308571429
Met	AUG	49	1

Val	GUU	48	0.684285714
	GUC	66	0.957142857
	GUA	29	0.375714286
	GUG^*∗*^	147	1.978571429

Ser	UCU	75	0.994285714
	UCC^*∗*^	118	1.862857143
	UCA	41	0.494285714
	UCG	55	0.832857143

Pro	CCU	117	0.85
	CCC^*∗*^	209	1.644285714
	CCA	108	0.735714286
	CCG	102	0.774285714

Thr	ACU	74	0.688571429
	ACC^*∗∗*^	208	2.145714286
	ACA	54	0.484285714
	ACG	82	0.684285714

Ala	GCU	84	0.792857143
	GCC^*∗*^	175	1.731428571
	GCA	71	0.655714286
	GCG	87	0.815714286

Tyr	UAU	35	0.54
	UAC	119	1.46

His	CAU	39	0.441428571
	CAC^*∗*^	116	1.558571429

Gln	CAA	62	0.537142857
	CAG^*∗*^	136	1.462857143^*∗*^

Asn	AAU	33	0.664285714
	AAC^*∗*^	72	1.335714286

Lys	AAA	41	0.598571429
	AAG^*∗*^	105	1.401428571

Asp	GAU	70	0.571428571
	GAC^*∗*^	210	1.428571429

Glu	GAA	72	0.592857143
	GAG^*∗*^	159	1.407142857

Cys	UGU	46	0.417142857
	UGC	135	1.582857143
Trp	UGG^*∗*^	128	1

Arg	CGU	45	0.532857143
	CGC^*∗∗*^	134	2.072857143
	CGA	54	0.667142857
	CGG	77	0.965714286

Ser	AGU	31	0.418571429
	AGC^*∗*^	91	1.395714286

Arg	AGA	63	0.752857143
	AGG^*∗*^	79	1.011428571

Gly	GGU	77	0.615714286
	GGC^*∗*^	219	1.738571429
	GGA	86	0.654285714
	GGG	124	0.991428571

TER	UAA	18	0.571428571
	UAG^*∗*^	20	1.538571429
	UGA	37	0.89

**Table 7:** Pearson relative analysis with GC_3S_.

	Pearson *r* (MMP-2)	*P* values (MMP-2)	Pearson *r* (MMP-9)	*P* values (MMP-9)
ENC	−0.993	0.000^*∗∗*^	−0.963	0.000^*∗∗*^
GC_12_	−0.851	0.015^*∗*^	−0.589	0.164

*Note.* ^*∗*^ *P* value < 0.05; ^*∗∗*^*p* value < 0.01; red represents strong correlation, blue represents moderate correlation, and black represents irrelevance.

## Data Availability

The yak *MMP-2* and *MMP-9* genes data used to support the findings of this study are included within the article and are available from the corresponding author upon request.
